# Molecular screening reveals non-uniform malaria transmission in western Kenya and absence of *Rickettsia africae* and selected arboviruses in hospital patients

**DOI:** 10.1186/s12936-022-04287-3

**Published:** 2022-09-17

**Authors:** Tatenda Chiuya, Jandouwe Villinger, Laura C. Falzon, Lorren Alumasa, Fredrick Amanya, Armanda D. S. Bastos, Eric M. Fèvre, Daniel K. Masiga

**Affiliations:** 1 International Centre of Insect Physiology and Ecology (icipe), P.O. Box 30772-00100, Nairobi, Kenya; 2 Department of Zoology and Entomology, University of Pretoria, Private Bag 20, Pretoria, 0028 South Africa; 3 Institute of Infection, Veterinary and Ecological Sciences, University of Liverpool, Leahurst Campus, Chester High Road, Neston, CH64 7TE UK; 4 International Livestock Research Institute, Old Naivasha Road, P.O. Box 30709, Nairobi, 00100 Kenya

**Keywords:** Malaria, Fever, Diagnosis, Prevalence, Socio-economic factors

## Abstract

**Background:**

In sub-Saharan Africa, malaria is the common diagnosis for febrile illness and related clinical features, resulting in the under-diagnosis of other aetiologies, such as arboviruses and *Rickettsia*. While these may not be significant causes of mortality in malaria-endemic areas, they affect the daily life and performance of affected individuals. It is, therefore, important to have a clear picture of these other aetiologies to institute correct diagnoses at hospitals and improve patient outcomes.

**Methods:**

Blood samples were collected from patients with fever and other clinical features associated with febrile illness at selected hospitals in the malaria-endemic counties of Busia, Bungoma, and Kakamega, and screened for Crimean-Congo haemorrhagic fever, Sindbis, dengue and chikungunya viruses, *Rickettsia africae*, and *Plasmodium* spp. using high-throughput real-time PCR techniques. A logistic regression was performed on the results to explore the effect of demographic and socio-economic independent variables on malaria infection.

**Results:**

A total of 336 blood samples collected from hospital patients between January 2018 and February 2019 were screened, of which 17.6% (59/336) were positive for *Plasmodium falciparum* and 1.5% (5/336) for *Plasmodium malariae.* Two patients had dual *P. falciparum*/*P. malariae* infections. The most common clinical features reported by the patients who tested positive for malaria were fever and headache. None of the patients were positive for the arboviruses of interest or *R*. *africae*. Patients living in Busia (OR 5.2; 95% CI 2.46–11.79; *p* < 0.001) and Bungoma counties (OR 2.7; 95% CI 1.27–6.16; *p* = 0.013) had higher odds of being infected with malaria, compared to those living in Kakamega County.

**Conclusions:**

The reported malaria prevalence is in line with previous studies. The absence of arboviral and *R. africae* cases in this study may have been due to the limited number of samples screened, low-level circulation of arboviruses during inter-epidemic periods, and/or the use of PCR alone as a detection method. Other sero-surveys confirming their circulation in the area indicate that further investigations are warranted.

**Supplementary Information:**

The online version contains supplementary material available at 10.1186/s12936-022-04287-3.

## Background

At least a hundred arboviruses, and several *Rickettsia* spp., are known to cause mild non-pathognomonic febrile illness in humans [1, 2], which in rare cases can lead to a range of severe complications such as encephalitis, haemorrhagic disorders, hepatitis, musculoskeletal impairment, and death [3]. However, some arboviruses, like Crimean-Congo haemorrhagic fever (CCHF), are associated with severe febrile illness and high fatality rates in humans [4]. The impact of the milder form of these diseases on human health is under-appreciated. However, studies show that they have notable impacts on the daily performance of affected individuals in terms of disability-adjusted life years (DALYS) [5]. Traditionally, arboviruses and *Rickettsia* are not considered significant causes of mortality and morbidity, especially in malaria-endemic resource-poor communities. Thus, funds allocated for their study and surveillance are limited [6], leading to misdiagnosis and poor assessment of their cumulative impact on community health [7, 8].

In the East African region, arboviruses are endemic, with the most important ones affecting humans being CCHF, Rift Valley fever, chikungunya, and dengue viruses [9]. Diverse tick-borne *Rickettsia* spp., particularly *Rickettsia africae*, have been widely reported in ticks collected from both the environment and cattle [10–13]; however, there is limited surveillance for these pathogens in the human population [14]. While few acute cases of spotted fever group rickettsiosis (SFGR), including African tick bite fever (caused by *R. africae*), have been reported in African indigenous people [15, 16], they are widely reported in travellers and expatriates visiting endemic areas in Africa. Indeed, SFGR are only second to malaria as a cause of illness in travellers returning from sub-Saharan Africa [17–19]. In East Africa, previous studies have shown that SFGR and arboviral illnesses contribute to non-malaria febrile illness in patients visiting hospitals [20–22]. Elsewhere in Africa, concurrent malaria and arboviral infections have also been documented [23, 24].

Western Kenya, comprising Busia, Bungoma and Kakamega counties, is situated at the border of Uganda and Kenya within the Lake Victoria basin of East Africa. Studies in this region have reported seropositivity to chikungunya [25], alphaviruses, flaviviruses [26, 27], phleboviruses (RVF) [28], and *Rickettsia* [14]. A single fatal human case of CCHF was also reported in this region [29]. In terms of malaria endemicity classification, Busia County is defined as a lake-endemic transmission zone (high stable transmission), while Bungoma and Kakamega counties have both lake-endemic and highland epidemic zones [30].

Great strides have been made in Kenya to study arboviral diseases during outbreaks in the known hotspots, such as the northern and coastal areas. However, fewer investigative efforts have been undertaken during inter-epidemic periods in those areas where clinical cases have been detected and have the potential to support circulation and outbreaks. Therefore, this study was undertaken in western Kenya using a subset of blood samples collected from patients with fever and other clinical features associated with febrile illness to determine the occurrence of acute cases of CCHF, Sindbis, dengue, and chikungunya viruses and *R. africae* in relation to malaria infection.

## Methods

### Study area

**Fig. 1:**
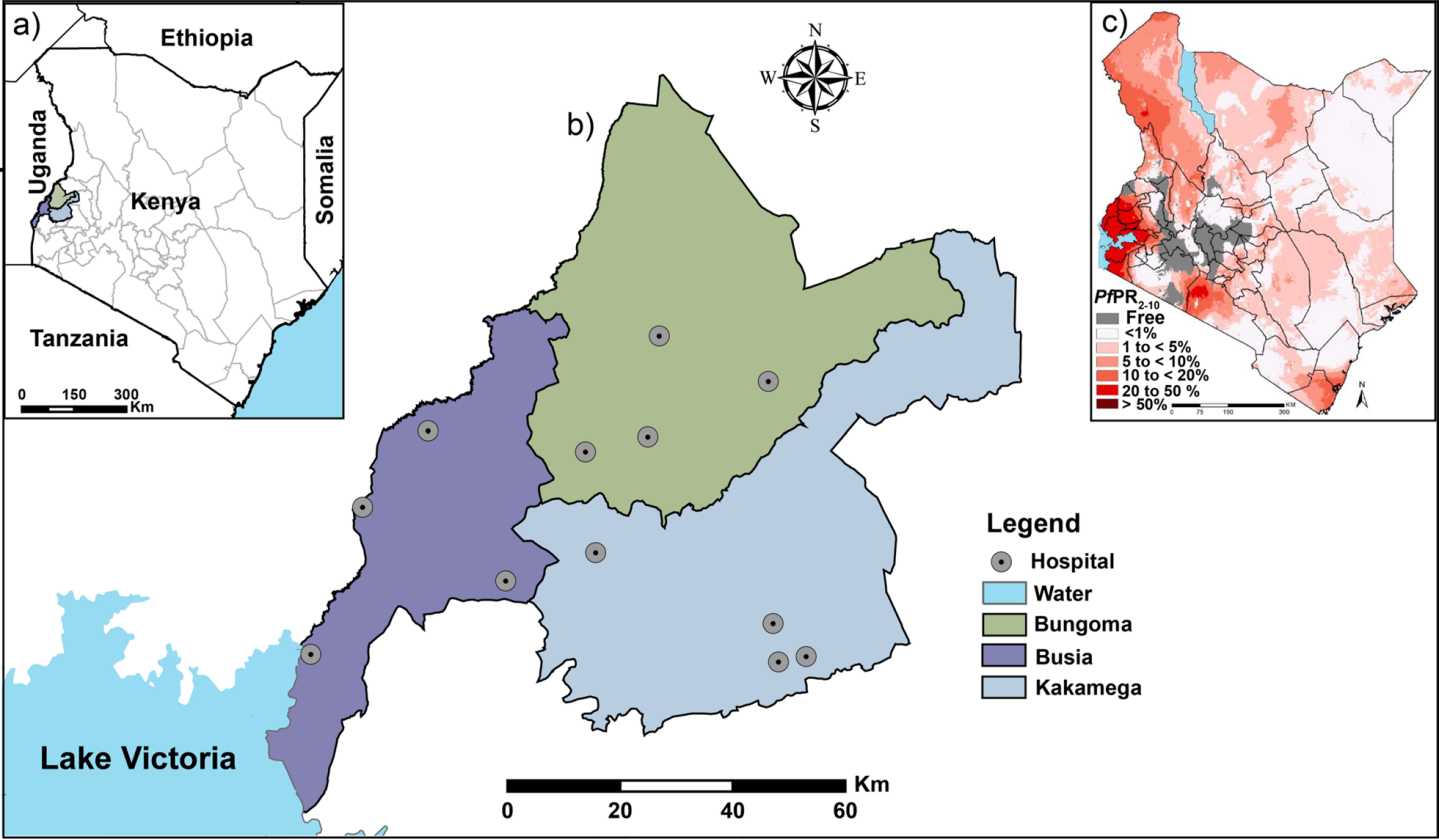
**Map of the study site in western Kenya**. **(a)** The highlighted three counties where the study was conducted, **(b)** the distribution of the hospitals from which patients were recruited and **(c)** malaria risk grading in Kenya based on the *Plasmodium falciparum* parasite prevalence data in children aged 2-10 years (PfPR2-10) [30]

This study was carried out in the three neighbouring counties of Busia, Bungoma, and Kakamega (Fig. 1a). Busia County lies on the shores of Lake Victoria and directly borders Uganda. Mt Elgon in Bungoma County is the highest point in the western region while the Kakamega rainforest is found in Kakamega County. The inhabitants of this region are mostly of the Luhya tribe. The economic activity in the area is centred around sugar cane farming and small holder livestock keeping in Bungoma and Kakamega County, while in Busia, fishing and also small holder livestock keeping is the major economic activity. Common to all the three counties is mixed subsistence farming characterized by intensification, diversification, and close integration of crop and livestock production [31]. The climate in this region is tropical with maximum temperatures ranging from 27 °C to 32 °C and rainfall ranging between 1,350 and 2,400 mm falling over two rainy seasons annually. These conditions favour the breeding of vectors and perennial malaria transmission [32]. The Busia-Uganda border is one of the busiest in East Africa with traffic transiting to Rwanda, Burundi, The Democratic Republic of Congo, and South Sudan. The human population has been estimated at 89,3681 in Busia, 1,671 million in Bungoma, and 1,868 million in Kakamega County [33].

### Study design and sampling procedure

The blood samples used in this study were collected as part of the Zoonoses in Livestock in Kenya (ZooLinK) project, as described in detail elsewhere [34]. The study aimed to develop and pilot a surveillance system for a number of zoonotic diseases in the area, involving collaborations between the animal and human health sectors for better diagnostics and enhanced awareness.

**Fig. 2:**
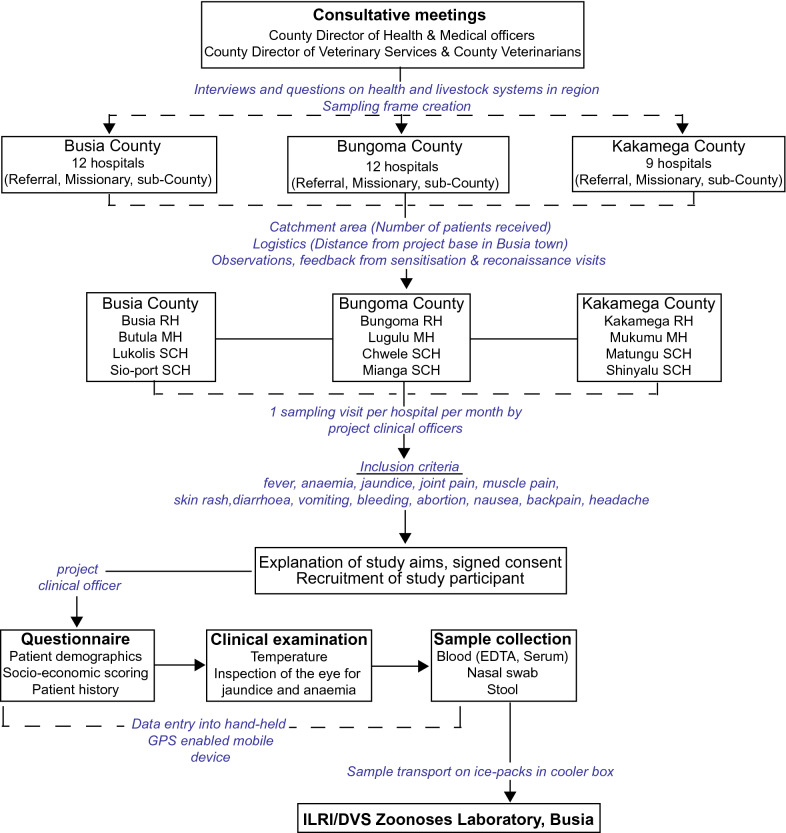
**Graphical illustration of the sampling framework at hospitals in western Kenya**. Based on Falzon et al. [34]. *RH* referral hospital; *MH* missionary hospital; *SCH* sub-County hospital; *ILRI* International Livestock Research Institute; *DVS* Department of Veterinary Services

Briefly, a sampling frame of 24 hospitals in the three counties was created, from which a total of 12 (4 per County) were selected for sampling. In each County, a referral hospital, a missionary hospital, and two sub-County hospitals were included in the study. Each of the selected hospitals was visited once every 4 weeks for 20 sampling cycles from 2017 to 2019. At each hospital, two project clinical officers liaised with the hospital staff to ensure smooth recruitment of study participants. Up to 10 participants were recruited at each hospital per visit. The inclusion criterion was patients presenting with one or more clinical features, including fever, suggestive of an arboviral/malaria/bacterial infection (Fig. 2). Fever, defined as a body temperature of  ≥ 37.5 °C was determined in patients by measuring axillary temperature with a digital thermometer. Self-reported fever was also inclusive even if the axillary temperature was within normal range.

Presence of jaundice and anaemia was determined by visual inspection of the sclera and palpebral conjunctiva. Muscle, joint, and back pain and other clinical features were captured as reported by the patients during history taking. Following signed consent, a brief questionnaire on current clinical features, current and previous treatments, and demographics, was administered. Blood samples were collected in 5-ml serum tubes and 4-ml EDTA tubes, which were then barcoded to maintain patient anonymity and confidentiality. Samples were transported to the field laboratory in Busia in a cooler box on ice packs and were later shipped on dry ice to the ILRI Nairobi laboratory where they were stored at −80 °C.

For the purpose of this study, 336 of 865 blood samples collected throughout the study, and associated demographic data were selected, based on the following criteria: (i) blood samples collected from patients with fever and other clinical features associated with febrile illness (Fig. 2) between January 2018 and February 2019, corresponding to the period ticks and mosquitoes positive for CCHF and Sindbis viruses, respectively, were collected from the same study site [10, 35], (ii) availability of complete meta-data, and (iii) adequate blood volume to conduct all analyses. Selected samples were thawed, aliquoted and transported on ice (0–5 °C) to the Martin Lüscher Emerging Infectious Disease (ML-EID) laboratory at the International Centre of Insect Physiology and Ecology (*icipe*) where nucleic acid extraction was performed on arrival and then stored at −80 °C for subsequent molecular analyses. The *icipe* ML-EID laboratory is located approximately 25 km (30–40 min) from the ILRI Nairobi laboratories.

### Nucleic acid extraction

The magnetic-based High Prep Viral DNA/RNA kit (Magbio Genomics Gaithersburg, USA), was used to isolate total nucleic acid from whole blood. Initially 200 µl of blood was added to 528 µl of a lysis master-mix consisting of VDR lysis buffer, isopropanol, and carrier RNA. After vortexing, 10 µl of proteinase K and 10 µl of MAG-S1 magnetic beads were added and mixed into solution by vortexing. The subsequent wash steps to separate protein and cellular debris from nucleic acid bound to magnetic particles were performed according to the manufacturer’s instructions. The High Capacity cDNA Reverse Transcription Kit (Life Technologies, Carlsbad, USA) was used to synthesize cDNA in 10 µl reactions according to the manufacturer’s instructions, supplementing the random primers with 600 µM non-ribosomal random hexanucleotide primers previously described for maximum yield [36].

### Detection of arboviruses and *Rickettsia africae*

To detect CCHF, Sindbis, dengue and chikungunya viruses in patients, a multiplex touchdown PCR and high-resolution melting analyses (PCR-HRM) were applied as an initial screening test to select positive samples for further identification, as described in detail by Villinger et al. [37] and Ajamma et al. [38] (for more details on the primers used for pathogen detection refer to Additional file 1). To detect the presence of *R. africae*, patients’ blood samples were screened with PCR-HRM using Rick-F1 and Rick-R2 primers that target the *Rickettsia* 16S rRNA region [12, 39].

### Detection of *Plasmodium spp*

Patients’ blood samples were screened for malaria-causing *Plasmodium* spp. using two sets of primers. Initially, a primer pair ncMS-F/ncMS-R was used to amplify a non-coding mitochondrial region (large subunit rRNA fragment E) of all *Plasmodium* spp. [40] using PCR-HRM [41]. All positive samples were selected by analysing melt and normalized profiles on Rotor-Gene Q software 2.1.0. Positive samples were further amplified using *cox* 1 primers targeting a 540-bp region of the cytochrome oxidase 1 gene of *Plasmodium* spp. [42]. *Plasmodium falciparum* DNA amplified and sequenced previously was included as a positive control in all the runs [43] (for more details on the thermocycling conditions refer to Additional file 1). Successful amplification was visualized by gel electrophoresis. Representative amplicons were purified by an Exo 1-rSAP combination (Biolabs, UK) and sent for bidirectional sequencing at Macrogen (Netherlands). Sequences were edited and cleaned using Geneious Prime version 2019.0.4 software (Biomatters, New Zealand). To confirm identity and relationship with previously described *Plasmodium* spp., nucleotide sequences were queried against the GenBank nr database using BLAST (Basic Local Alignment Search Tool).

### Data analyses

Logistic regression to evaluate the determinants of malaria infection was performed in R^®^ version 4.0.3 using the generalized linear model (GLM) for binary responses. The independent variables included baseline socio-economic, demographic, and geographic variables such as county of residence, sex, age, occupation, floor type in house, livestock ownership, use of mosquito nets, and education level (for more details on the management of independent variables for statistical analysis refer to Additional file 2). Malaria prevalence based on a positive PCR test result for *Plasmodium* spp. was estimated for each of the independent variable categories. Odds ratios, confidence intervals, and *P*-values were estimated, with a *P* ≤ 0.05 being considered statistically significant.

## Results

### Socio-economic and demographic characteristics of study participants

Table 1 shows the socio-economic and demographic profile of the 336 patients whose blood samples were analysed in this study. Of the 529 samples excluded from this study, 338 had inadequate volume for analysis, 162 were collected before January 2018, and 29 had incomplete metadata. Amongst the depleted blood samples (n = 338), 306 were above 13 years of age. Fever as a clinical feature (n = 214) was measured in 70 of the participants and self-reported in the other 144 patients. Overall, 37% (124/336) of the participants were from Bungoma County, 35% (117/336) from Kakamega, and 28% (95/336) from Busia County. Twenty-six percent (87/336) of the participants were males compared to 74% (249/336) females. The age of the participants ranged from 6 to 88 years; most were aged between 10–19 years (27%; 90/336) or 50 years and above (23%; 77/336). Farming was the most common occupation (106/336; 31.5%), while students contributed an equal proportion of the study participants (105/336; 31.3%). Ninety-two percent (309/336) of the participants reported coming from a household that owned livestock. Ninety-six percent (324/336) of the participants reported having at least one mosquito bed-net at home. Only 6% (20/336) of the participants came from a household where the female head/spouse had not received any level of formal education, while 29.5% (99/336) came from a household where the female head/spouse had gone up to form 4 and beyond.

**Table 1:** Socio-economic and demographic variables of the patients recruited for this study by county

Characteristic	Busia county		Bungoma county		Kakamega county		Combined	
	N	%	N	%	N	%	N	%
Total	95		124		117		336	
Sex								
Male	23	24.2	35	28.2	29	24.8	87	25.9
Female	72	75.8	89	71.8	88	75.2	249	74.1
Age (years)								
0–9	5	5.3	2	1.6	5	4.3	12	3.6
10–19	33	34.7	33	26.6	24	20.5	90	26.8
20–29	18	18.9	17	13.7	30	25.6	65	19.3
30–39	10	10.5	19	15.3	18	15.4	47	14
40–49	12	12.6	20	16.1	13	11.1	45	13.4
50 +	17	17.9	33	26.6	27	23.1	77	22.9
Occupation								
Farmer	28	29.5	44	35.5	34	29.1	106	31.5
Trader	13	13.7	15	12.1	19	16.2	47	14
Student	37	38.9	37	29.8	31	26.5	105	31.3
Other	6	6.3	20	16.1	16	13.7	42	12.5
Unemployed	11	11.6	8	6.5	17	14.5	36	10.7
Floor type								
Mud/wood	48	50.5	75	60.5	62	53	185	55.1
Cement/tiles	47	49.5	49	39.5	55	47	151	44.9
Livestock ownership								
Yes	86	90.5	112	90.3	111	94.9	309	92
No	9	9.5	12	9.7	6	5.1	27	8
Mosquito nets								
Yes	92	96.8	119	96	113	96.6	324	96.4
No	3	3.2	5	4	4	3.4	12	3.6
Education level								
None	9	9.5	3	2.4	8	6.8	20	5.9
Class 1–7	24	25.3	22	17.7	27	23.1	73	21.7
Class 8 & Form 1–3	39	41	58	46.8	47	40.2	144	42.9
Form 4 & above	23	24.2	41	33.1	35	29.9	99	29.5

### *Plasmodium* spp., arbovirus, and *Rickettsia* detection

**Fig. 3:**
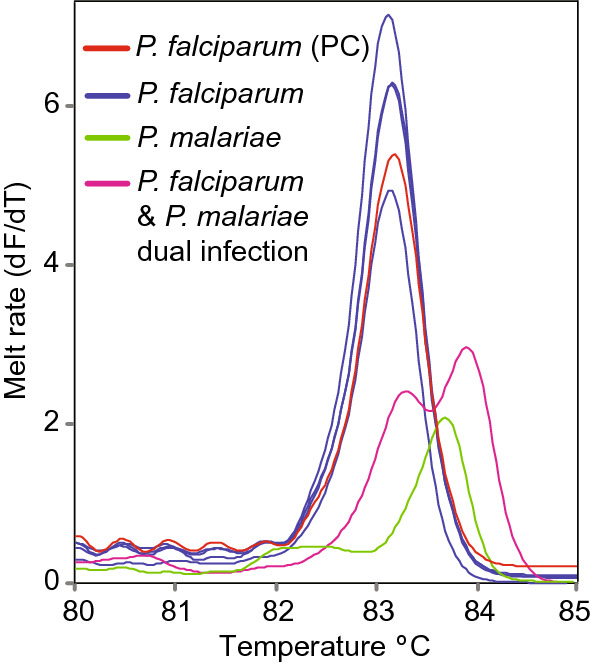
Melt rate profiles of positive representative samples. *Plasmodium falciparum*, *P. malariae* and *P. falciparum/P. malariae* mixed infections based on amplification of the non-coding mitochondrial gene (nc-MS) of *Plasmodium* spp. are shown. *PC* positive control

All patient blood samples tested negative for CCHF, Sindbis, dengue and chikungunya viruses and *R. africae*. However, by PCR-HRM analysis, *P. falciparum* (GenBank accessions MT430947-MT430947) and *Plasmodium malariae* (GenBank accession MT430946) infections were detected in patients presenting to selected hospitals in the counties under study. Two patients had dual *P. falciparum*/*P. malariae* infections (Fig. 3).

### Malaria prevalence, univariable and multivariable logistic regression analyses

The overall prevalence of malaria in the patients that were recruited in this study at hospitals in the three neighbouring counties was 19.6% (66/336), as determined by a positive PCR result for *P. falciparum* or *P. malariae.* Specifically, the prevalence was 17.6% for *P. falciparum* and 1.5% for *P. malariae*. The prevalence of malaria in the different independent variable categories is shown in Table 2. Busia County had the highest prevalence (32.6%) followed by Bungoma County (20.2%), while Kakamega County had the lowest (8.5%). Overall, more females were recruited into the study, compared to male participants; nonetheless, the malaria prevalence was higher in males. The prevalence of malaria was highest in those patients who were between 0 and 9 years of age. Most of the participants came from farming households followed by those who reported as being students; however, the malaria prevalence was higher in the latter group, possibly confounded by age. The participants were equally divided into those who reported living in a house with a mud/wood floor and those living in a house with a cement/tile floor, with a higher prevalence of malaria in the latter group. The prevalence of malaria was higher in patients that reported having no mosquito net, compared to those that had one at home. Similarly, the prevalence was higher in patients who came from households without livestock, compared to those who had livestock at home. Malaria prevalence was highest in patients who came from a household where the female head or spouse did not have any formal education, compared to the other education categories.

**Table 2:** Univariable logistic regression of malaria infection in patients at hospitals in western Kenya

Variable	Categories	Prevalence %	Odds ratio (95% CI)	*P*-value
County				
	Busia	31/95 (32.6)	5.2 (2.46–11.79)	< *0.001*
	Bungoma	25/124 (20.2)	2.7 (1.27- 6.16)	*0.013*
	Kakamega	10/117 (8.5)	Ref.	Overall: < *0.001*
Sex				
	Male	21/87 (24)	1.4 (0.79–2.57)	0.222
	Female	45/249 (18.1)	Ref.	
Age (years)				
	0–9	5/12 (41.7)	3.2 (0.85–11.63)	0.075
	10–19	24/90 (26.7)	1.6 (0.79–3.51)	0.195
	20–29	11/65 (16.9)	0.92 (0.38–2.18)	0.844
	30–39	5/47 (10.6)	0.5 (0.16–1.52)	0.263
	40–49	7/45 (15.6)	0.82 (0.29–2.18)	0.711
	50 +	14/77 (18.2)	Ref.	Overall: 0.096
Occupation				
	Unemployed	9/36 (25)	1.6 (0.63–3.98)	0.292
	Trader	5/47 (10.6)	0.6 (0.18–1.57)	0.315
	Student	27/105 (25.7)	1.69 (0.87–3.35)	0.124
	Other	7/42 (16.7)	1 (0.35–2.46)	0.963
	Farmer	18/106 (17)	Ref.	Overall: 0.169
Floor type				
	Mud/wood	33/185 (17.8)	0.8 (0.45–1.33)	0.357
	Cement/tiles	33/151 (21.9)	Ref.	
Livestock ownership				
	Yes	60/309 (19.4)	0.8 (0.34–2.38)	0.725
	No	6/27 (22.2)	Ref.	
Mosquito nets				
	No	5/12 (41.7)	3.1 (0.89–9.97)	0.062
	Yes	61/324 (18.8)	Ref.	
Education level				
	None	8/20 (0.4)	4 (1.38–11.69)	*0.01*
	Class 1–7	15/73 (20.5)	1.57 (0.7–3.53)	0.27
	Class 8 & Form 1–3	29/144 (20.1)	1.53 (0.77–3.15)	0.231
	Form 4 & above	14/99 (14.1)	Ref.	Overall: 0.09

Statistically significant *p*-values are shown in italics

The most common clinical features in the patients who tested positive for malaria were fever and headache, followed by abdominal cramps, joint pain, and back pain in decreasing order. There were fewer occurrences of the other clinical features as reported by the patients.

On univariable logistic regression analysis, county of residence, age, “mosquito nets” and education level were marginally associated with the occurrence of malaria, while the other independent variables were not (Table 2). Therefore, multivariable logistic regression analysis was carried out on the association of these four variables and malaria infection.

The multivariable model contained only ‘county of residence’ as the variable significantly associated with malaria infection; therefore, no final model is presented as this was the same as that presented for the univariable analysis (Table 2). Age, “mosquito nets”, and education level were sequentially dropped as their association with malaria infection in the multivariable analysis was not significant. At each stage the resultant model was compared to the preceding model using the likelihood ratio test. Malaria prevalence was higher in Busia (OR 5.2; 95% CI 2.46–11.79; *p* < 0.001) and Bungoma (OR 2.7; 95% CI 1.27–6.16; *p* = 0.013) counties, compared to Kakamega County (Table 2). These estimated odds ratios indicate 5- and 3-times higher odds of having malaria in the patients residing in the counties of Busia and Bungoma counties, respectively, than in those from Kakamega County.

## Discussion

Co-circulation of malaria, arboviruses and/*Rickettsia* infections in malaria endemic areas can result in misdiagnoses, as they share the same clinical syndromes and diagnostic testing for the latter two pathogens is not routinely available. This study, reports on the prevalence of malaria in patients that presented to hospitals in western Kenya with fever and other symptoms suggestive of an arboviral or *Rickettsia* infection. However, CCHF, Sindbis, dengue and chikungunya viruses and *R. africae* were not detected. The study clearly shows that, while malaria is an important cause of febrile illness and other associated clinical features in this region, overall, it was present in only 17% of the patients. This means that, besides the pathogens investigated in this study, further work is still required to understand other causes of febrile illness and associated clinical features.

The prevalence of malaria in Busia County was less than that reported previously in the same area around the Lake Victoria shores (37%) [44], in Kisumu (38.3%) [32] and in Homa bay County (45.8%) [45]. These studies however, mostly focused on and reported malaria infection only in children ranging between 6 months and 15 years, rather than in patients of all ages. In malaria endemic regions with stable transmission, malaria infection is higher in children compared to adults due to a gradual age-dependent build-up of immunity [46]. This is also evident in this study where the prevalence of malaria was 41.7% in children under 9 years but comparatively lower in the older age groups. Comparable malaria prevalences were reported in other studies in patients on Mfangano and neighbouring islands on Lake Victoria in Homa Bay County [47, 48]. Higher prevalence of malaria in Busia and Bungoma, compared to Kakamega County, concurs with past reports of heterogeneity in transmission patterns in western Kenya, with higher transmission around the Lake Victoria shores in Busia being reported compared to other areas [44]. In all these previous studies, the reported malaria prevalence determined by PCR was consistently higher than that by microscopy or rapid diagnostic test (RDT), affirming the higher sensitivity of PCR in comparison to the latter tests. The complications that can arise because of this misdiagnosis, which include improper treatment options, drug wastage and confounding of malaria prevalence estimates, have been extensively reviewed before [48, 49].

Overall, the counties of Kisumu, Siaya, Migori, Homa Bay, Kakamega, Busia, Bungoma, and Vihiga are classified as having lake endemic malaria transmission [44]. However, the western Kenyan region where this study was carried out has both stable and epidemic malaria transmission. This is because among these eight counties, Bungoma, Kakamega and Vihiga do not share a border with Lake Victoria hence they have pockets of epidemic malaria transmission resulting in lower malaria prevalence. Stable transmission is characterized by hyper endemic transmission rates, with an average of one infective bite/person/week throughout the year, while epidemic transmission occurs in less prevalent zones where the population has less immunity [30].

The most frequent species in this study was *P. falciparum* as reported by other studies with less occurrence of *P. malariae* and *Plasmodium ovale* [32, 47, 48]. Most of the clinical features that were reported by the patients who tested positive for malaria are generally shared between rickettsial/arboviral illnesses, highlighting the difficulty of deriving a correct diagnosis for these infections based on clinical signs and symptoms alone without further laboratory testing.

The other variables in this study were not significantly associated with malaria infection. Bed net possession was high among the participants; however, its correct and consistent use was not assessed. The association between bed nets and malaria prevalence may be masked in some studies, as some patients get infective bites before retiring to bed or early morning due to the changing ecology and behaviour of the mosquito vector [50], improper use of the bed nets [51], and aging of the nets themselves [52].

Patient age is usually an important predictor in higher transmission settings, with infants under the age of 5 years being at a higher risk [53]. However, in low transmission settings, the risk will also extend into adulthood [32, 54]. The effect of sex has not been fully elucidated, with previous studies showing conflicting findings. Women were more likely to be infected than males in Kisumu [55]. Socio-cultural activities expose both women and men to infective mosquito bites, albeit in different ways [56].

The effect of livestock at households on malaria transmission is dynamic, with the consensus that livestock can zoo-potentiate transmission when close to or inside human dwellings [57–59], but can also act as zoo-prophylaxis when placed a distance away from households [60]. In this study, the proximity of livestock to the homesteads was not established. Floor-type was used as a proxy to determine the socio-economic status of the households in which the patients lived. Higher socio-economic status is usually associated with improvement in the housing conditions and consequently less predisposition to malaria [61–63]. This association may be confounded by the fact that most of the proxies for socio-economic status are in essence more directly related to malaria exposure and risk [64].

This study attempted to determine acute arboviral and rickettsial infections in relation to malaria infection, as has been reported before in Kisumu County [22], Homa Bay County [65], and Asembo [14, 66] in western Kenya, and in Tanzania [21]. However, outside outbreak phases, circulation of arboviruses is very low and it is therefore challenging to detect clinical/acute cases [22, 67]. This is also reflected in the low infection rates of the vector mosquito species from the same region, where only one pool of *Culex* mosquitoes was positive for Sindbis virus, in combination with a comparatively high infection rate with insect-specific flaviviruses [35]; the latter are thought to block superinfection of vectors with pathogenic viruses [68]. On the other hand, *Plasmodium* spp.-chikungunya dual infection has been shown to cause interferon gamma induced suppression of viraemia and joint pathology caused by arthralgic alphaviruses [69]. This is plausible in this study given that this is a malaria endemic region where most people are likely to be asymptomatic carriers [70], and the most sero-prevalent arbovirus detected previously in this area was chikungunya [25].

Due to the low detection of arboviral infections by PCR, mostly because of a brief viraemic period and low viral load, previous studies in the same region have tried to highlight the circulation of arboviruses in human populations and/or determine their contribution to febrile illness by serology [25, 27, 71]. However, serological tests are time consuming due to the need to measure different antibodies (IgA/IgM/IgG) and their titers at several stages of illness to distinguish between an active infection and past exposure [27]. Additionally, these tests have low specificity as they are prone to cross-reactivity in populations where several *alphaviruses* and *flaviviruses* are endemic [25, 72]. The problem of cross-reactivity can be alleviated by performing plaque reduction neutralization tests, which usually require at least BSL-2 facilities. Therefore, the standard recommendations for the detection of both arboviruses and *Rickettsia* involve a combination of direct pathogen detection (culture, antigen, or nucleic acid) and detection of a fourfold rise in antibody titers between acute- and convalescent-phase sera [73, 74]. In this study, some cases may have been missed due to the afore-mentioned limitations, that are associated with the use of PCR alone. Future studies combining the use of serology and PCR may be required to generate more informative conclusions on the occurrence of arboviral disease and rickettsioses. In this case, serology could not be performed due to serum sample constraints.

While previous studies have described high infection rates of *Amblyomma variegatum* ticks with *R. africae* [10], the findings in this study suggest a lack of significant widespread rickettsaemia in humans and livestock. If actively circulating in humans, the prevalence is likely to be extremely low, requiring very large sample sizes to be accurately quantified. The use of archived blood samples for this study may have affected the recovery and detection of pathogens of interest by PCR in the blood due to several freeze–thaw cycles. To counter the occurrence of false negatives, internal PCR controls that amplify host messenger RNA can be developed and used in future viral analysis. The other limitation of the study was that the subjects were recruited as part of a larger surveillance study to determine the occurrence of zoonotic diseases (including arboviral) in hospital patients. Therefore, subjects were selected on the basis of inclusion criteria that did not solely consider fever, hence the absence of standard non-malarial febrile illness definition and assessment of anaemia for some of the subjects. Additionally, some of the hospitals that were included had a separate children’s clinic, which could not be sampled simultaneously, resulting in the enrolment of comparatively fewer children. Further, the malaria infection described in this study was based on PCR, which can detect even asymptomatic malaria carriers. This means that their clinical signs and symptoms could have been caused by other concurrent infecting pathogens that were not detected. It is, therefore, important in future studies to compare PCR based diagnosis *versus* microscopy and RDT as it highlights the advantages and limitations of each method.

## Conclusions

This study reports on the occurrence of malaria and associated risk factors in western Kenya in patients wherein it also sought to determine other causes of fever and related clinical features such as arboviruses and *Rickettsia.* While arboviruses or *Rickettsia* were not detected, they may be contributing a proportion of the febrile illness, at levels too low to be detected without very large studies. This presumption is on the backdrop of the reported circulation of arboviruses and *Rickettsia* by several serological studies in the same study region. The presence of livestock in the participants’ households is a variable of interest as reports on zoo-potentiation and zoo-prophylaxis are still conflicting. It is also an important factor in the control of arbovirus-transmitting mosquitoes, which are more zoophilic compared to malaria vectors. Distinguishing the proximity of the livestock to households is, therefore, of paramount importance in future studies. Only the ‘county of origin’ was a significant determinant of malaria infection in this study, highlighting the regions/counties of priority in malaria prevention programmes. This information is important in the implementation of targeted interventions.

## Supplementary Information

**Additional file 1. **Detailed description of primers and thermocycling conditions used for the detection of *Rickettsia africae*, arboviruses and *Plasmodium* spp.

**Additional file 2. **Detailed description of the management of independent variables for statistical analysis.

## Data Availability

The dataset generated and analysed in this study can be made available from the corresponding authors on reasonable request. All the nucleotide sequences generated from this study have been deposited and are available in the GenBank database under the accession numbers indicated in text.

## Abbreviations

OR: Odds ratio
CCHF: Crimean-Congo haemorrhagic fever
DALYS: Disability adjusted life years
IREC: Institutional Research Ethics Committee
ZooLinK: Zoonoses in livestock in Kenya
P*f*PR_2-10_: *Plasmodium falciparum* parasite prevalence data in children aged 2–10 years
ILRI: International Livestock Research Institute
NACOSTI: National Commission for Science, Technology and Innovation
EDTA: Ethylene diamine tetra acetic acid
ML-EID: Martin lüscher emerging infectious disease
*icipe*: International centre of insect physiology and ecology
DVS: Department of veterinary services
MH: Missionary hospital
SCH: Sub-County hospital
RH: Referral hospital
DNA: De-oxy ribonucleic acid
RNA: Ribonucleic acid
cDNA: Copy DNA
PCR-HRM: Polymerase chain reaction-high resolution melting
rRNA: Ribosomal RNA
GLM: Generalized linear model
RDT: Rapid diagnostic test
RT-PCR: Reverse transcriptase-polymerase chain reaction
IgM: Immunoglobulin M
SFGR: Spotted fever group rickettsioses
CI: Confidence interval
