# Supplementary material for: Molecular screening reveals non-uniform malaria transmission in western Kenya and absence of Rickettsia africae and selected arboviruses in hospital patients
Source: Malar J. 2022 Sep 17;21:268. doi: 10.1186/s12936-022-04287-3 (PMC9482282; doi:10.1186/s12936-022-04287-3)
Supplement: Supplementary file 1 — Additional file 1. Detailed description of primers and thermocycling conditions used for the detection of Rickettsia africae, arboviruses and Plasmodium spp. [file 12936_2022_4287_MOESM1_ESM.docx]

**Additional file 1:** Detailed description of primers and thermocycling conditions used for the detection of *Rickettsia africae*, arboviruses and *Plasmodium* spp.

*Detection of arboviruses*

The individual primers in the multiplex reaction were optimised to detect *flavi*-, *nairo*- and *alpha*-viruses. The “*Flavi JV*” primers targeted the NSP5 region of *flaviviruses* while the “*Nairo L*” targeted the L-segment of *nairoviruses*. The “*Vir 2052*” primers were used to detect *alphaviruses* targeting the NSP4 region. Each of the primer pairs generated a 150 bp fragment for high resolution melting analysis (HRM). The reactions were performed in a Rotor-Gene Q HRM thermo-cycler (Qiagen Hilden, Germany). The HRM products were purified using an Exo 1-rSAP combination (Biolabs, UK) and identified after sequencing (Macrogen, Netherlands).

*Table showing the target genes and primer sequences for arboviral, R. africae and Plasmodium spp. detection*

| Target gene | Primer name | Primer sequence (5’-3’) |
| --- | --- | --- |
| *Flavivirus* NSP5 | Flavi JV2a F | AGYMGHGCCATHTGGTWCATGTGG |
|  | Flavi JV2b F | AGCCGYGCCATHTGGTATATGTGG |
|  | Flavi JV2c F | AGYCGMGCAATHTGGTACATGTGG |
|  | Flavi JV2d F | AGTAGAGCTATATGGTACATGTGG |
|  | Flavi JV2a R | GTRTCCCADCCDGCDGTRTCATC |
|  | Flavi JV2b R | GTRTCCCAKCCWGCTGTGTCGTC |
| *Nairovirus* L-segment | Nairo L 1a F | TCTCAAAGATATCAATCCCCCCITTACCC |
|  | Nairo L 1b F | TCTCAAAGACATCAATCCCCCTTWTCCC |
|  | Nairo L 1a R | CTATRCTGTGRTAGAAGCAGTTCCCATC |
|  | Nairo L 1b R | GCAATACTATGATAAAAACAATTMCCATCAC |
|  | Nairo L 1c R | CAATGCTGTGRTARAARCAGTTGCCATC |
|  | Nairo L 1d R | GCAATGCTATGGTAGAAACAGTTTCCATC |
|  | Nairo L 1e R | CRAKGCTGTGGTAAAAGCAGTTRCCATC |
| *Alphavirus* NSP4 | Vir 2052 F | TGGCGCTATGATGAAATCTGGAATGTT |
|  | Vir 2052 R | TACGATGTTGTCGTCGCCGATGAA |
| *Rickettsia* 16S rRNA | Rick-F | GAACGCTATCGGTATGCTTAACACA |
|  | Rick-R | CATCACTCACTCGGTATTGCTGGA |
| *Plasmodium* ncMS | ncMS-F | TAGCCGACAAGGAATTTTGC |
|  | ncMS-R | CCTTGAATGGAGCACTGGAT |
| *Plasmodium* *cox* 1 | COX1-F | AGAACGAACGCTTTTA ACGCCTG |
|  | COX1-R | ACTTAATGGTGGAT ATAAAGTCCATCCWGT |

*Detection of Rickettsia africae*

For the detection of *R. africae*, the PCR mixtures contained 2 µl of 5X HOT FIREPol EvaGreen HRM mix (Solis BioDyne, Estonia), 0.5 µM of each forward and reverse primer and 2 µl of template DNA in a final reaction volume of 10 µl. *Rickettsia africae* DNA amplified and sequenced in a previous analysis in our lab was used as a positive control in each run. Thermocycling and HRM analysis were carried out in a Rotor-Gene Q HRM thermo-cycler (Qiagen Hilden, Germany) using previously described conditions.

*Reaction mixtures and cycling conditions for the detection of Plasmodium* spp*.*

The reaction mixture for the initial screening for *Plasmodium* spp. using the ncMS-F/ncMS-R primer pair was made up of 2 µl of 5X HOT FIREPol EvaGreen HRM mix (Solis BioDyne, Estonia), 0.5 µM of each forward and reverse primer and 1 µl of template DNA. Thermal cycling was carried out in a Rotor-Gene Q HRM thermo-cycler (Qiagen Hilden, Germany) and conditions were set as follows: initial denaturation at 95°C for 15 min, followed by 40 cycles of denaturation at 95°C for 20 sec., annealing at 61°C for 15 sec and extension at 72°C for 20 sec. There were five post-cycling hold steps, at 72°C for 7 min, 95°C for 15 sec., 68°C for 1 min., 80°C for 15 sec., and 60°C for 15 sec. The 190-bp amplicons were melted from 75°C to 90°C with 0.1°C increments. For further identification of *Plasmodium* spp. using the *cox* 1 primers, PCR reactions containing 7.5 µl of 2X MyTaq HS Mix (Bioline, UK), 0.5 µM of each forward and reverse primer, 2 µl of template DNA were performed in a final reaction volume of 15 µl. Thermo-cycling was carried out in a SimpliAmp thermo-cycler (Applied biosystems, Singapore) with the following set conditions: initial denaturation at 95°C for 3 min followed by 35 cycles of denaturation at 95°C for 20 sec., annealing at 59°C for 30 sec and extension at 72 °C for 30 sec.
