# Supplementary material for: Molecular screening reveals non-uniform malaria transmission in western Kenya and absence of Rickettsia africae and selected arboviruses in hospital patients
Source: Malar J. 2022 Sep 17;21:268. doi: 10.1186/s12936-022-04287-3 (PMC9482282; doi:10.1186/s12936-022-04287-3)
Supplement: Supplementary file 2 — Additional file 2. Detailed description of the management of independent variables for statistical analysis. [file 12936_2022_4287_MOESM2_ESM.docx]

**Additional file 2:** Detailed description of the management of independent variables for statistical analysis

The independent variables before statistical analysis in R^®^ version 4.0.3 were managed as follows: County of residence (Busia/Bungoma/Kakamega), sex (male/female), and mosquito net use (YES/NO) were used in the analyses as collected from the study participants. However, age, recorded in years was recoded into six categories (*0-9*, *10-19*, *20-29*, *30-39*, *40-49* and *50 and above*) before analysis. Occupation was re-categorized into farmer, trader, student, unemployed and “*other*”. The latter category included occupations such as teacher, police, tailor, security guard, motorcycle rider, driver, and shopkeeper. Floor type was recoded into two levels: patients who reported living in a house with a mud or wooden floor, and those in a house with a cement or tiled floor. Livestock ownership was recoded into two categories; “*YES*” if patients reported coming from a household that had goats, sheep, chickens, pigs, /and/or cattle, or “*NO*” for those that reported not having any livestock. Education level in this study was specific to the female head of the household or female spouse where the patient lived and was recoded into four categories; “*none*” if she did not have any formal education, “*Class 1-7*”, “*Class 8 & Forms 1-3*”, and “*Form 4 & above*”. The Kenyan education curriculum has 8 years of basic education/primary school) (Class 1-8), 4 years of secondary school (Form 1-4) and thereafter 4 years of undergraduate studies.
